# Upfront combination therapy with sildenafil and ambrisentan in patients with chronic thromboembolic pulmonary hypertension

**DOI:** 10.36416/1806-3756/e20250056

**Published:** 2025-11-14

**Authors:** William Salibe-Filho, Tulio Martins Vieira, José Leonidas Alves-Junior, Yally Priscila Pessôa Nascimento, Luiza Sarmento Tatagiba, Caio Julio Cesar Fernandes, Carlos Viana Poyares Jardim, Mario Terra-Filho, Rogerio Souza

**Affiliations:** 1. Divisão de Pneumologia, Instituto do Coração, Hospital das Clínicas, Faculdade de Medicina, Universidade de São Paulo, São Paulo (SP) Brasil.

**Keywords:** Pulmonary Embolism, Drug therapy, combination, Hypertension, pulmonary

## Abstract

**Objective:**

Chronic thromboembolic pulmonary hypertension (CTEPH) is a rare complication of acute pulmonary embolism, being characterized by persistent obstruction of pulmonary vessels and leading to increased pulmonary vascular resistance and right ventricular failure. Although pulmonary endarterectomy is the preferred treatment, medical therapies may offer clinical benefits in specific settings. We sought to evaluate the clinical and hemodynamic response of CTEPH patients treated with sildenafil and ambrisentan upfront combination therapy.

**Methods:**

This was a retrospective cohort study including patients with operable and inoperable CTEPH. The patients were followed from 2019 to 2022 and were treated with sildenafil and ambrisentan as first-line therapy.

**Results:**

Functional and hemodynamic data were analyzed at baseline and after a minimum of six months of therapy. Following treatment, there was a notable improvement in functional class, natriuretic peptide levels, and invasive hemodynamics.

**Conclusions:**

The combined use of sildenafil and ambrisentan appears to be associated with clinical, functional, and hemodynamic improvement in patients with CTEPH.

## INTRODUCTION

Chronic thromboembolic pulmonary hypertension (CTEPH) is a rare complication of acute pulmonary embolism,^1^ being characterized by chronic obstruction of the pulmonary vasculature by organized thrombi and leading to increased pulmonary vascular resistance (PVR) and right ventricular failure.^2^ Progressive remodeling of the distal pulmonary arteries and arterioles^3^ may also occur. This is due to several factors, including high pulmonary vascular pressure and shear stress associated with persistent fibrothrombotic remodeling, local inflammation, and circulating vascular mediators.^3^ ^,^ ^4^ These changes are similar to those observed in patients with pulmonary arterial hypertension (PAH), including intimal thickening and plexiform lesion development.^5^ These changes lead to progressive vascular remodeling, which modifies vascular endothelial cell responses, compromises fibrinolysis, and affects annexin expression^6^ ^,^ ^7^ and heat shock protein regulation,^7^ ultimately causing vascular disruption in patients with CTEPH. 

The preferred treatment for CTEPH is pulmonary endarterectomy (PEA), balloon pulmonary angioplasty (BPA) being the treatment of choice for inoperable patients.^8^ ^,^ ^9^ However, mechanical interventions are not always feasible, being dependent on thrombus accessibility, coexisting comorbidities, and patient preference.^10^ For ineligible patients, medical therapies represent an option for clinical and hemodynamic improvement. In patients with inoperable CTEPH, the use of bosentan (an endothelin receptor antagonist) has been reported to reduce PVR without significantly affecting the six-minute walk distance (6MWD).^11^ Conversely, the use of riociguat (an oral stimulator of soluble guanylate cyclase) has been reported to increase the 6MWD by 36 m in inoperable patients and in patients with residual PAH following PEA.^12^ In a phase 2 study, macitentan (an endothelin receptor antagonist) was shown to have a positive effect on the 6MWD.^13^ A prospective study of ambrisentan was discontinued in 2019 because of low recruitment.^14^

There is substantial evidence to support the use of medical therapies in selected patients with CTEPH; however, the role of different treatment strategies has yet to be addressed in this setting. In patients with PAH, upfront combination therapy has become the standard of care,^15^ ^,^ ^16^ which is largely due to the results of a study comparing the effects of combined therapy with ambrisentan and tadalafil against monotherapy and demonstrating the superiority of dual oral therapy.^17^ In patients with CTEPH, the five-year survival rate for those receiving combination therapy has been reported to be similar to that of those receiving monotherapy.^18^ However, there is a lack of data regarding the use of combination therapy as an upfront treatment strategy. 

The objective of the present study was to evaluate the clinical and hemodynamic response of CTEPH patients treated with sildenafil and ambrisentan upfront combination therapy. 

## METHODS

This was a retrospective cohort study of patients followed at our institution-a referral center for CTEPH management-from 2019 to 2022. Because of the COVID-19 pandemic, the number of PEA procedures was drastically reduced. Given the clinical or hemodynamic severity, medical therapy was initiated during the evaluation of a potential surgical intervention at the discretion of the attending physician, with patients being classified as operable or inoperable at the time of medical treatment initiation. The diagnosis of CTEPH was based on established guidelines.^19^ The inclusion criterion was having received upfront combination therapy with sildenafil and ambrisentan. The study variables included functional class, B-type natriuretic peptide (BNP) levels, and hemodynamic data from right heart catheterization, collected before and after a minimum of six months of treatment with sildenafil and ambrisentan. The present study was approved by the local research ethics committee (Protocol no. CAAE 11032919.8.0000.0068). 

The Wilcoxon signed-rank test was used in order to compare continuous variables before and after medical therapy. Fisher’s exact test was used in order to compare categorical variables. Data distribution was tested for normality with the Kolmogorov-Smirnov test. Continuous variables were expressed as median and interquartile range. All statistical analyses were performed with GraphPad Prism software, version 9.0 (GraphPad Software, Inc., San Diego, CA, USA), with values of p < 0.05 being considered significant. 

## RESULTS

A total of 32 CTEPH patients receiving sildenafil and ambrisentan were included in the present study, with a mean age of 50 years. Most of the patients were in functional class III or IV, showing severe hemodynamic impairment (Table 1). All patients were started on sildenafil at a dose of 20 mg three times a day; however, during the follow-up period the dose was increased at the discretion of the attending physician. At the follow-up evaluation, 13 patients were receiving 20 mg of sildenafil three times a day; 11 were receiving 40 mg three times a day; 5 were receiving 60 mg three times a day; and 3 were receiving 80 mg three times a day. All patients were concurrently treated with ambrisentan at a dose of 10 mg/day. 

**Table 1:** Baseline characteristics of the study population.^a^

Characteristic	
	Baseline (N = 32)
Female	17 (53.1)
Age, years	50.3 ± 14.9
Anticoagulant, DOAC	18 (56.2)
NYHA functional class	
I	0
II	4 (12.5)
III	22 (68.7)
IV	6 (18.7)
BNP, pg/dL	337.5 [140.3-603.5]
Hemodynamic parameters	
RAP, mmHg	17.0 [11.5-20.0]
PAOP, mmHg	11.5 [8.0-15.0]
mPAP, mmHg	59.5 [52.5-64.7]
Cardiac output, L/min	2.9 [2.1-3.9]
PVR, dyn • s^−1^ • cm^−5^	1,258 [832-1,558]

DOAC: direct oral anticoagulant; NYHA: New York Heart Association; BNP: B-type natriuretic peptide; RAP: right atrial pressure; PAOP: pulmonary artery occlusion pressure; mPAP: mean pulmonary artery pressure; and PVR: pulmonary vascular resistance. ^a^Data presented as n (%), mean ± SD, or median [IQR].

**Figure 1:**
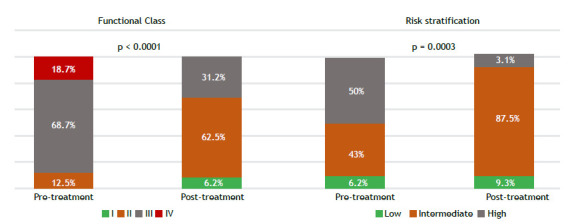
Pre- and post-treatment functional class and risk stratification.

**Figure 2:**
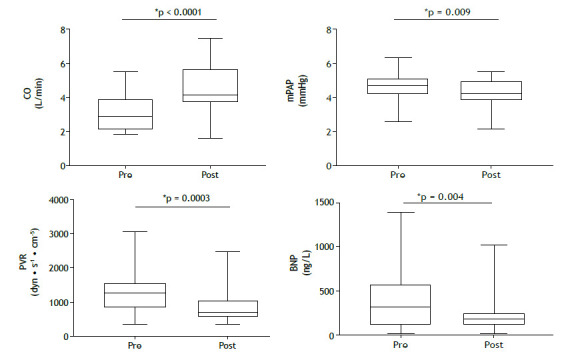
Pre- and post-treatment evaluation of hemodynamics (n = 32) and B-type natriuretic peptide (BNP) levels (n = 26). CO: cardiac output; mPAP: mean pulmonary artery pressure; and PVR: pulmonary vascular resistance.

After a median treatment follow-up of 13.2 months (IQR, 10-22), we observed a significant improvement in functional class, with the proportion of patients in functional class III or IV decreasing from 87.5% to 31.2% (p < 0.001). Prior to treatment, 50% of the patients were classified as being high-risk patients. Following treatment, only 3.10% remained in the high-risk category, with the majority transitioning to intermediate risk (Figure 1). This was accompanied by a significant decrease in BNP levels and an improved hemodynamic profile (Figure 2 and Table 2), including a 38% reduction in PVR, driven by a 43% increase in cardiac output, a 7% decrease in mean pulmonary artery pressure (mPAP; Figure 2), and a 29% reduction in right atrial pressure (Table 2). All patients underwent hemodynamic assessment before and after treatment. BNP levels were available for all patients at baseline but only for 26 at the follow-up evaluation. Neither rehabilitation nor angioplasty was performed. No severe side effects were observed during the follow-up period. 

**Table 2:** Pre- and post-treatment evaluation.^a^

	Pre-treatment	Post-treatment	p-value
BNP, ng/L (n = 26)	426.9 [140.3-603.5]	186 [109-292.8]	p = 0.0004
RAP, mmHg	17.0 [11.5-20.0]	12.0 [8.0-17.2]	p = 0.009
CO, L/min	2.9 [2.1-3.9]	4.1 [3.7-5.6]	p < 0.0001
mPAP, mmHg	59.5 [52.5-64.7]	53.5 [48.2-62.0]	p = 0.009
PVR, dyn • s^-^¹ • cm^-5^	1,258 [832-1,558]	718.5 [528-1,034]	p = 0.0003

BNP: B-type natriuretic peptide; RAP: right atrial pressure; CO: cardiac output; mPAP: mean pulmonary artery pressure; and PVR: pulmonary vascular resistance. ^a^Data presented as median [IQR].

## DISCUSSION

Our study demonstrated that patients with CTEPH experienced significant clinical and hemodynamic improvement following upfront combination therapy with sildenafil and ambrisentan. To the best of our knowledge, this is the first study to investigate the role of ambrisentan used in combination with sildenafil in this context. 

In an international registry of patients in Europe and Canada,^20^ 50.1% of the patients were male, with a mean age of 63 years. Hemodynamic results showed an mPAP of 47 mmHg, a PVR of 709 dyn • s^−1^ • cm^−5^, and a cardiac index of 2.2 L • min^−1^ • m^−2^.^20^ Similarly, in a study reporting results from the United Kingdom National Cohort,^21^ the mean age was 57 years, with 53% of the patients being male. Hemodynamic findings included a mean baseline mPAP of 47 mmHg and a PVR of 830 dyn • s^−1^ • cm^−5^.^21^ Although our patient population was younger, they presented with more severe hemodynamic impairment and were therefore selected for medical treatment prior to PEA. 

Recently, a worldwide CTEPH registry demonstrated that the use of targeted medical therapy prior to mechanical intervention was significantly more common before BPA (63%) than before PEA (25%).^22^ The most frequently used medications were phosphodiesterase type 5 inhibitors and endothelin receptor antagonists, although without a description of their combined use.^22^ Our study introduced this therapeutic approach as a potential strategy for patients with CTEPH. 

Medical therapy as a bridge to PEA has been shown to delay the surgical procedure without clear evidence of improved patient outcomes.^23^ However, this might not apply to patients with severe hemodynamic impairment at diagnosis. In a trial of BPA vs. riociguat for the treatment of inoperable CTEPH,^8^ riociguat administered before BPA in patients with higher hemodynamic impairment was associated with fewer adverse events, highlighting the potential benefit of medical therapy in more severe cases. At the 7th World Symposium on Pulmonary Hypertension, the proposed treatment algorithm included medical therapy prior to BPA for patients with mPAP ≥ 40 mmHg or PVR > 4 Wood units,^24^ further emphasizing the importance of medical therapy in patients with significant hemodynamic impairment. Nevertheless, combination therapy for CTEPH, particularly as upfront treatment and including operable patients, remains a poorly explored area. Despite evidence supporting combination strategies in patients with PAH, data for patients with CTEPH are scarce and mostly limited to inoperable cases or monotherapy trials.^18^ In a previous study, our group demonstrated that patients with CTEPH and a preoperative cardiac output of < 3.75 L/min had poorer postoperative outcomes.^25^ In such cases, medical therapy was associated with improved overall survival after PEA, which justified the use of combination therapy in our cohort to optimize hemodynamics for future surgical interventions. 

A recent study evaluating the use of selexipag in inoperable patients with CTEPH or patients with residual PAH after PEA was discontinued because of futility. The trial failed to demonstrate a treatment effect on the primary endpoint of PVR.^26^ Ambrisentan had previously been tested in the same setting, showing a trend of improvement in the 6MWD and a reduction in PVR as a secondary endpoint.^14^ However, the study was terminated early because of low enrollment.^14^ In a study published in 2013,^12^ riociguat resulted in a significant (31%) reduction in PVR of 226 dyn • s^−1^ • cm^−5^. In a trial assessing the use of bosentan,^11^ there was a 24.1% reduction in PVR, although without improvement in the 6MWD, a coprimary endpoint of the study. Similarly, in a phase II study assessing the use of macitentan exclusively in inoperable patients,^13^ ^,^ ^27^ there was a reduction of 206 dyn • s^−1^ • cm^−5^ (16%) in the treatment group and a reduction of 86 dyn • s^−1^ • cm^−5^. (8%) in the control group, the efficacy and safety of macitentan being also demonstrated in the extension study.^27^ In our study, patients receiving dual therapy showed a 38% reduction in PVR, corresponding to a reduction of 494 dyn • s^−1^ • cm^−5^. and exceeding the aforementioned reductions. This finding is consistent with the hemodynamic effects of combination therapy in patients with PAH, such as those observed in a study demonstrating a reduction of approximately 50% in PVR with dual or triple upfront therapy.^28^ Our findings raise the question of the most appropriate strategy to be employed when medical therapy is considered in patients with CTEPH, a topic that warrants thorough investigation in future prospective trials. 

Other key findings in our study include improvements in functional class and BNP levels, which are consistent with those of other studies.^11^ ^,^ ^12^ One of the aforementioned studies^11^ showed a 622 ng/L reduction in N-terminal pro-BNP levels, whereas the other^12^ showed a 291 ng/L reduction, both being consistent with our observed improvements in BNP levels and functional class.^11^ ^,^ ^12^

Our study has several limitations that must be acknowledged. First, the study was conducted at a single center, although it is the largest center for CTEPH management in Brazil. Second, given the constraints imposed by the COVID-19 pandemic, patients could not be classified as operable or inoperable prior to initiation of medical therapy, the decision of initiating medical treatment being solely based on clinical and hemodynamic severity, thus potentially creating a selection bias for treating the most severe cases with upfront combination therapy. Nevertheless, patients with more severe hemodynamic profiles are the most likely to benefit from a more aggressive therapeutic approach prior to mechanical intervention. Third, the fact that six-minute walk tests were not regularly performed during the COVID-19 pandemic prevented an analysis of the impact of the sildenafil-ambrisentan combination on the exercise capacity of patients. Although riociguat remains the only approved medical therapy for CTEPH, its unavailability in our region justified the use of sildenafil and ambrisentan in the present study. Another limitation of the present study is the absence of a standardized protocol for sildenafil dosing, which may have influenced the hemodynamic response to medical treatment. Finally, during follow-up, only a few of the patients undergoing PEA underwent invasive hemodynamic assessment after surgery, which prevented an analysis of the potential benefit of the sildenafil-ambrisentan combination on surgical outcomes. In addition, this was a retrospective observational study without a control group. Being a before-and-after analysis, it is subject to several biases such as missing data and nonstandardized documentation. Ideally, therapeutic efficacy should be evaluated in a prospective randomized controlled trial. 

In conclusion, patients with CTEPH showed significant clinical, functional, and hemodynamic improvement with the combined use of sildenafil and ambrisentan as medical therapy. Our findings suggest that this combination may be a valuable addition to the treatment strategy for CTEPH and should be further evaluated in future prospective studies. 
